# Comparative Efficacy and Safety of Different Tenecteplase Doses With Alteplase in Acute Ischemic Stroke: A Systematic Review With Pairwise and Network Meta‐Analysis to Determine the Optimal Dose

**DOI:** 10.1002/brb3.70756

**Published:** 2025-08-19

**Authors:** Muhammad Hassan Waseem, Zain ul Abideen, Muhammad Haris Khan, Muhammad Fawad Tahir, Marium Khan, Hafsa Arshad Azam Raja, Ameer Haider Cheema, Sania Aimen, Muhammad Arslan Tariq, Javed Iqbal

**Affiliations:** ^1^ Allama Iqbal Medical College Lahore Pakistan; ^2^ King Edward Medical University Lahore Pakistan; ^3^ Saidu Medical College Swat Pakistan; ^4^ H.B.S Medical and Dental College Islamabad Pakistan; ^5^ Jinnah Sindh Medical University Karachi Pakistan; ^6^ Rawalpindi Medical University Rawalpindi Pakistan; ^7^ UT Southwestern Medical Center Texas USA; ^8^ Quetta Institute of Medical Sciences Quetta Pakistan; ^9^ Nursing Department Hamad Medical Corporation Doha Qatar

**Keywords:** Alteplase, dose analysis, network meta‐analysis, pairwise, stroke, thrombolytic therapy, tenecteplase

## Abstract

**Background:**

Tenecteplase (TNK) is a novel thrombolytic agent gaining attention as an alternative to alteplase for treating acute ischemic stroke (AIS). This meta‐analysis evaluates the safety and efficacy of various TNK doses compared to alteplase, integrating recent randomized controlled trials (RCTs) and employing a frequentist network meta‐analysis to identify the optimal dose while addressing existing evidence gaps.

**Methods:**

Up until December 2024, PubMed, Cochrane Central, and ScienceDirect were searched. Using Review Manager 5.4.1 for pairwise meta‐analysis, the Risk Ratios (RR) with 95% Confidence Intervals (CI) were pooled under the random effects model. Additionally, R version 4.3.2 and the “netmeta” package were used to conduct a network meta‐analysis for various dosages of the two thrombolytic drugs.

**Results:**

Thirteen RCTs, pooling 9,044 patients, were included in the quantitative synthesis. TNK was associated with a statistically significant improvement in the excellent functional outcome (mRS 0–1) (RR = 1.04; 95% CI: [1.00, 1.08]; p = 0.03) compared to alteplase. No significant differences were observed between TNK and alteplase in terms of good functional outcome (mRS 0–2), poor functional outcome (mRS 5–6), major neurological improvement within 72 h, symptomatic intracranial hemorrhage (sICH), or mortality at 90 days. On network analysis, TNK 0.25 mg/kg showed significant improvement in excellent functional outcome (RR = 1.05, 95% CI: [1.01, 1.10]) and TNK 0.32 mg/kg in good functional outcome (1.30, 95% CI: [1.15, 1.48]) compared to alteplase. According to the P‐score ranking, TNK 0.25 mg/kg was ranked as the best for achieving an excellent outcome (P‐score = 0.86), and TNK 0.1 mg/kg as the worst (P‐score = 0.16). For symptomatic intracranial hemorrhage (sICH), alteplase 0.9 mg/kg was ranked as the best (P‐score = 0.71), and TNK 0.4 mg/kg as the worst (P‐score = 0.17).

**Conclusion:**

TNK (0.25‐0.32 mg/kg) demonstrates superior efficacy compared to alteplase in achieving functional outcomes for AIS, while alteplase remains safer regarding sICH. Optimal dosing favors TNK 0.25 mg/kg for efficacy, but safety considerations highlight the need for individualized thrombolytic selection.

## Graphical abstract

This study analyzes data from 13 RCTs involving over 9000 patients, comparing tenecteplase (TNK) doses with alteplase for acute ischemic stroke (AIS). It shows significant functional improvements with TNK (0.25‐0.32 mg/kg), while safety endpoints (sICH, mortality) are comparable to alteplase, guiding clinicians in selecting the optimal thrombolytic and dosage for AIS treatment.

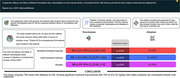

## 1 Introduction

Acute ischemic stroke (AIS) happens when a blood clot blocks blood flow to the brain, leading to the rapid loss of neurological function. Being one of the leading causes of death and permanent disability, stroke remains a significant global health concern. (Laurent et al. 2024). There were 101.5 million people worldwide affected by strokes in 2019, including 77.2 million cases of AIS. Ischemic stroke represents 87% of all strokes in the United States, whereas intracranial hemorrhage (ICH) and subarachnoid hemorrhage (SAH) make up 10% and 3%, respectively (Virani et al. 2021; Virani et al. 2020). Early intervention is crucial, as it significantly reduces morbidity and improves functional recovery (Laurent et al. 2024). Conventional treatment for AIS includes the use of intravenous thrombolytic agents to dissolve clots and restore cerebral perfusion (Bhaskar et al. 2018). For many years, the accepted course of treatment has been alteplase, a recombinant tissue plasminogen activator (tPA), which is typically administered within 4.5 h of symptom onset. Despite its proven benefits, alteplase has limitations, including a relatively short half‐life and an increased risk of symptomatic ICH (sICH) (Wang et al. 2023).

TNK has emerged as a potential alternative to alteplase for AIS treatment. A genetically altered tPA called TNK has several pharmacological benefits, including a longer half‐life that permits single‐bolus administration and increased fibrin specificity, which may lead to improved clot dissolution (Wang et al. 2024). These characteristics could make TNK more convenient in emergencies and enhance its efficacy. TNK is also believed to improve recanalization and reperfusion while maintaining a low risk of hemorrhage and may be more effective at breaking down large vessel clots (Campbell et al. 2018; Campbell et al. 2015).

Although several meta‐analyses have compared TNK with alteplase, many have either omitted dose‐specific analyses necessary for identifying the optimal dose or have not included the latest high‐quality randomized controlled trials (RCTs), which limit their clinical applicability. Our study fills these existing literature gaps by integrating newly published trials, including the ORIGINAL (Meng et al. 2024) and ATTEST‐2 (Muir et al. 2024) trials, and using a frequentist network meta‐analysis to assess and rank the efficacy and safety of various TNK doses. This method enables a more detailed evaluation of thrombolytic agents in AIS, including the selection of optimal dose, providing clearer, evidence‐based guidance for informed clinical decision‐making.

## 2 Methods

This meta‐analysis adhered to the Preferred Reporting Items for Systematic Reviews and Meta‐Analyses (PRISMA) guidelines (Page et al. 2021) and followed the reporting standards and procedures described in the Cochrane Handbook for Systematic Reviews of Interventions (Higgins et al. 2019). This review's protocol was pre‐registered on PROSPERO under the **ID CRD42024605380**.

### 2.1 Literature Search and Search Strategy

PubMed, Cochrane Central, and ScienceDirect were searched until December 2024 for studies comparing the drug TNK with alteplase for AIS. The terms “Tissue Plasminogen Activator,” “Tenecteplase,” “Alteplase,” and “Ischemic Stroke” were utilized as medical subject headings (MeSH). Additionally, the bibliographies of included studies were searched to obtain pertinent data. Supplementary Table S1 shows the articles received from the comprehensive search strategies in each database.

### 2.2 Selection Criteria

**FIGURE 1:**
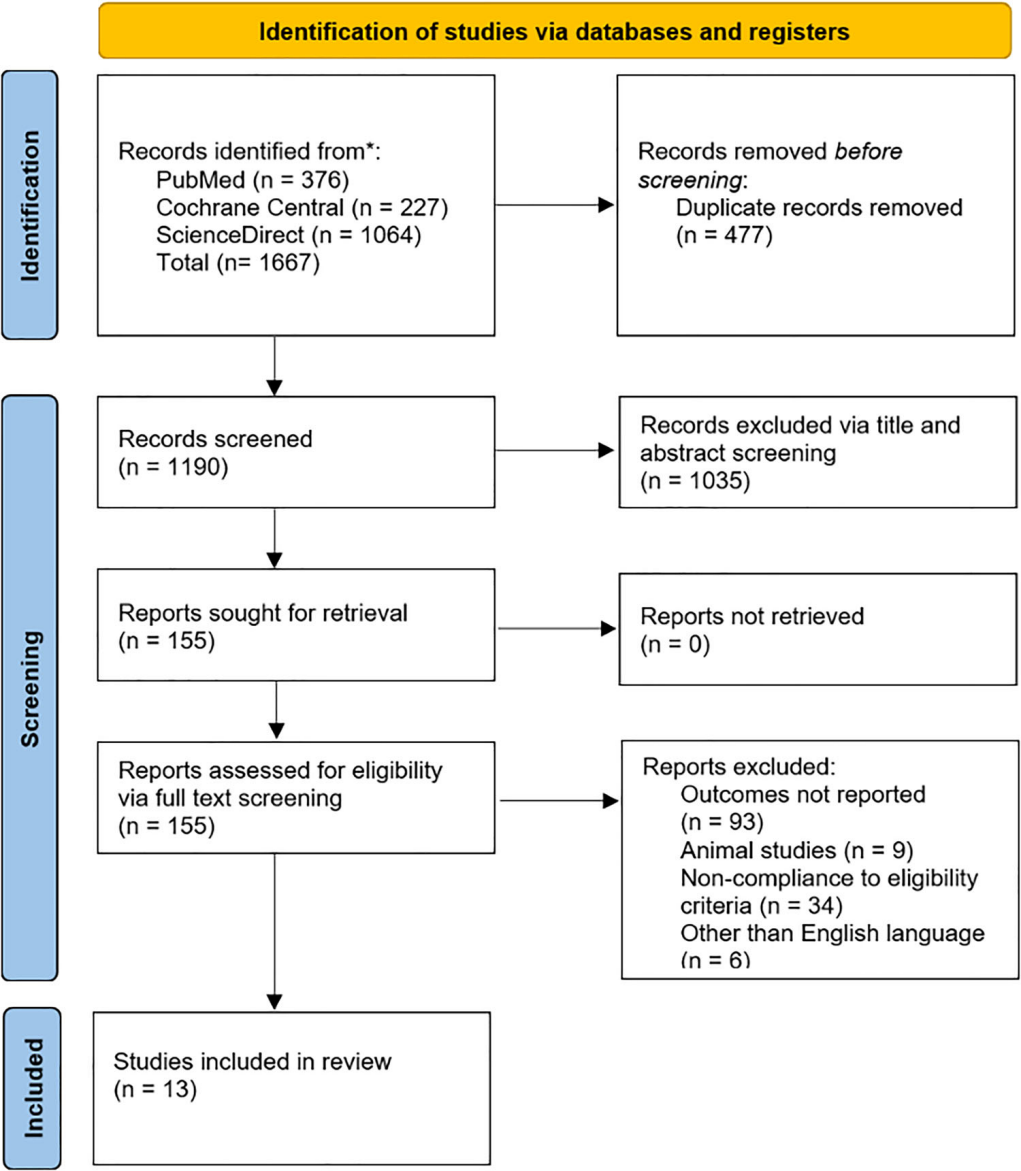
PRISMA flowchart of the study selection process.

All articles obtained from the initial search were imported into EndNote version 20. After duplicate removal, two authors (M.F.T. and M.H.K.) reviewed the remaining articles independently, undergoing title and abstract screening. After the exclusion based on the primary screening, the remaining articles were examined against predetermined criteria to assess their eligibility. The studies that were included adhered to the following criteria: (1) Population: adults aged over 18 years experiencing AIS within 4.5 h, (2) Intervention: patients treated with TNK at various doses, including TNK 0.25 mg/kg, TNK 0.1 mg/kg, TNK 0.32 mg/kg, and TNK 0.4 mg/kg, (3) Comparator: Alteplase 0.9 mg/kg, (4) Outcomes: An excellent functional outcome (modified Rankin Scale (mRS) 0–1) at 90 days was the primary outcome of interest. The secondary outcomes analyzed were mortality at 90 days, good functional outcome (mRS 0–2) at 90 days, poor functional outcome (mRS 5–6) at 90 days, major neurological improvement within 72 h, and sICH, and (5) Study Design: RCTs. The studies excluded were those that did not report TNK or Alteplase, articles written in languages other than English, study designs such as cross‐sectional studies, cohorts, commentaries, reviews, and research that did not include AIS patients. A PRISMA flowchart, as shown in Figure 1, illustrates the study selection process.

### 2.3 Data Extraction

Two authors (Z.U.A. and M.K.) used a Microsoft Excel sheet (Microsoft Corp., Redmond, WA) to extract data from studies that met the inclusion criteria. Data regarding baseline study characteristics and outcomes were extracted. The conflict was resolved by consultation with the senior author (M.H.W.). The extracted baseline data included the author's name, location, study design, sample size (*n*), mean age (SD), intervention and control dosages, percentage of males, prior stroke, current smokers, median National Institutes of Health Stroke Scale (NIHSS) score, and occlusion location. The primary outcome of interest was the excellent functional outcome at 90 days. Secondary outcomes included 90‐day mortality, good and poor functional outcomes at 90 days, major neurological improvement within 72 h, and sICH. Functional outcomes were assessed using the mRS scale, a standard clinical tool for evaluating disability or dependence in stroke patients. Higher scores on this scale, which goes from 0 to 6, indicate higher levels of disability. Major neurological improvement is defined as either an NIHSS score of 0–1 at 24–72 h or a decrease of 8 points or more in the NIHSS score from baseline. Exceptions include the NOR‐TEST (Logallo et al. 2017), NOR‐TEST 2 (Kvistad et al. 2022), and ORIGINAL (Meng et al. 2024) trials, where a decrease of 4 points or more in NIHSS from baseline is used as a metric to define major neurological improvement.

### 2.4 Evaluation of Bias

The Cochrane Risk of Bias tool (RoB 2.0) (Sterne et al. 2019) was used to evaluate the risk of bias in the RCTs. This involves assessing bias across five domains, including bias resulting from the actual randomization process, bias due to deviations from the planned intervention, bias caused by missing outcome data, bias related to outcome assessment, and bias arising from the selection of reported outcomes. All included studies were subjected to this assessment, and their biases were categorized as low, some concerns, and high. Independent review and examination of bias among studies were conducted by two authors (M.A.A and S.A). A senior author (M.H.W) was consulted to address disagreements on evaluation. The publication bias was assessed statistically using Egger's regression and graphically using funnel plots. We performed the GRADE assessment of all the outcomes using the GRADEpro GDT (Guyatt et al. 2011) to determine the certainty of evidence.

### 2.5 Statistical Analysis

The statistical analysis was performed on the Review Manager software version 5.4.1 (RevMan n.d.). The Mantel‐Haenszel random effects model was used to pool the risk ratios (RR) alongside a 95% confidence interval (CI) for the dichotomous outcomes. The forest plots were used to illustrate the pooled estimates of effect sizes. Heterogeneity was evaluated by the Cochrane Q test and Higgins' inconsistency (I^2^) statistics (Higgins et al. 2003). When heterogeneity exceeded 40%, a leave‐one‐out sensitivity analysis was performed to identify its cause. The *p*‐value of < 0.05 was deemed statistically significant. Additionally, a frequentist model network meta‐analysis was conducted to determine the optimal dose of TNK using R software version 4.3.2. The “meta” and “netmeta” packages were utilized. The relative ranking of the different thrombolytic doses was determined using the P‐score. The results of the network meta‐analysis were presented as network evidence plots. We also conducted node‐splitting analysis to identify the inconsistencies between direct and indirect evidence for each outcome, with a p‐value of < 0.05 considered statistically significant. The network estimates for different comparisons were presented as league tables.

## 3 Results

### 3.1 Search Results

We retrieved 1667 articles from databases such as PubMed, ScienceDirect, and Cochrane Library. After eliminating duplicates (*n* = 477), we were left with 1190 articles that underwent title and abstract screening, resulting in 155 articles. These were then subjected to full‐text screening, resulting in 13 RCTs being included in the final quantitative analysis. (Meng et al. 2024; Muir et al. 2024; Logallo et al. 2017; Kvistad et al. 2022; Parsons et al. 2024; Wang et al. 2023; Menon et al. 2022; Bivard et al. 2022; Li et al. 2022; Campbell et al. 2018; Huang et al. 2015; Parsons et al. 2012; Haley et al. 2010).

### 3.2 Included Studies Characteristics

This quantitative synthesis pooled a total of thirteen RCTs (Meng et al. 2024; Muir et al. 2024; Logallo et al. 2017; Kvistad et al. 2022; Parsons et al. 2024; Wang et al. 2023; Menon et al. 2022; Bivard et al. 2022; Li et al. 2022; Campbell et al. 2018; Huang et al. 2015; Parsons et al. 2012; Haley et al. 2010). These studies, published between 2010 and 2024, encompassed 9044 patients. The sample size ranged from 75 to 1777, while the mean age ranged from 64.4 to 73 years. Individuals with prior stroke episodes ranged from 2% to 24%. The median NIHSS score across the included studies ranged from 4 to 17 for the TNK and alteplase treatment arms. The internal carotid artery is the most occluded in these patients with AIS. Three studies were conducted in China (Meng et al. 2024; Wang et al. 2023; Li et al. 2022), four in Australia (Parsons et al. 2024; Bivard et al. 2022; Campbell et al. 2018; Parsons et al. 2012), and two in Norway (Logallo et al. 2017; Kvistad et al. 2022). The remaining studies were conducted in the United States, the United Kingdom, Scotland, New Zealand, Belgium, Finland, Canada, Spain, and Taiwan. The doses of TNK administered were 0.1 mg/kg (*n* = 116), 0.32 mg/kg (*n* = 60), 0.4 mg/kg (*n* = 668), and 0.25 mg/kg (*n* = 3788). The included trials utilized a 0.9 mg/kg dose of alteplase (*n* = 4413). Table 1 presents the baseline characteristics of the included studies.

**TABLE 1:** Baseline characteristics of the included studies.

					Male %					Baseline NIHSS score, Med(IQR)		Occlusion location		
Study ID	Location	Study design	Sample Size (n)	Mean age, yr (SD)	**TNK**	**Alteplase**	Dosage	Prior stroke n (%) (TNK/Alteplase)	Current smoke n (%) (TNK/Alteplase)	**TNK**	**Alteplase**	**ICA %**	**MCA**	**MCA**
													**M1 %**	**M2%**
TRACE 2 2023 (Wang et al., 2023)	China	RCT	1417	65.5	492 (69%)	479 (68%)	TNK (0.25mg/kg)	—	266 (38%) / 276 (39%)	7 (5–10)	7 (6–10)	—	—	—
				−10.7			Alteplase							
							(0.9 mg/ kg)							
Act 2022 (Menon et al., 2022)	Canada	RCT	1577	73	424 (52·6%)	398 (51·6%)	TNK (0.25 mg/kg)	—	—	9 (6–16)	10 (6–17)	86.6	15.21	22.52
				−15.2			Alteplase							
							(0.9 mg/ kg)							
TASTE‐A 2022 (Bivard et al., 2022)	Australia	RCT	104	72.9	33 (60%)	30 (61%)	TNK	5 (9%) / 9 (18%)	8 (15%) / 9 (18%)	8 (5–14)	8 (5–17)	—	—	—
				−17.9			(0.25 mg/kg)							
							Alteplase							
							(0.9 mg/ kg)							
NOR‐TEST 2 2022 (Kvistad et al. 2022)	Norway	RCT	204	71.2	45 (45·0%)	53 (51·0%)	TNK	17 (17·0%) / 10 (9·6%)	24 (24%) / 25 (24%)	11·5 (8–17)	11 (8–17·5)	—	—	—
				−13.9			(0.4 mg/ kg)							
							Alteplase (0.9 mg/kg)							
TRACE 2021 (Li et al., 2022)	China	RCT	236	64.4	132 (74.6%)	38 (64.4%)	TNK	—	71 (40.1%)/ 24 (40.7%)	8.0 (5.0‐12.0)	8.0 (5.0–12.0)	—	—	—
				−12.1			(0.1 mg/ kg, 0.25 mg/kg, 0.32 mg/kg)							
							Alteplase							
							(0.9 mg/ kg)							
EXTEND‐IA TNK 2018 (Campbell et al., 2018)	Australia and New Zealand	RCT	202	71.15 (14.4)	58 (57%)	52 (51%)	TNK (0.25 mg/kg)	—	—	17 (12–22)	17 (12–22)	23.7	58.9	14.35
							Alteplase							
							(0.9 mg/ kg)							
NOR‐TEST 2017 (Logallo et al. 2017)	Norway	RCT	1100	71	321 (58%)	339 (62%)	TNK	119 (22%)/120 (22%)	169 (31%) / 177 (32%)	4 (2–7)	4 (2–8)	—	—	—
				−13.8			(0.4 mg/ kg)							
							Alteplase (0.9 mg/kg)							
ATTEST 2015 (Huang et al., 2015)	Scotland	RCT	96	71	30 (64%)	31 (63%)	TNK (0.25 mg/kg)	12 (26%)/11 (22%)	13 (28%) / 10 (20%)	12 (9–18)	11 (8–16)	24.6	42.4	23.2
				−12.4			Alteplase							
							(0.9mg/ kg)							
Parsons 2012 (Parsons et al., 2012)	Australia	RCT	75	70	26 (52%)	12 (48%)	TNK	—	14 (28%) / 1 (4%)	*14.6±2.3	14.0±2.3*	1.33	28	8
				−8.3			(0.1 mg/ kg, 0.25 mg/kg)							
							Alteplase							
							(0.9 mg/ kg)							
Haley	USA	RCT	111	69.1	41 (51%)	17 (51%)	TNK	21 (25.9%)/4 (13%)	9 (11%) / 7 (23%)	10 (6–15)	13 (5–17)	—	—	—
2010 (Haley et al., 2010)				−16.5			(0.1 mg/ kg, 0.25 mg/kg, 0.4 mg/kg)							
							Alteplase							
							(0.9 mg/ kg)							
ORIGINAL 2024 (Meng et al. 2024)	China	RCT	1465	65.3	517 (70.6%)	502 (68.5%)	TNK	14 (1.9%)/13 (1.8%)	—	6 (5.0‐8.5)	6 (5.0‐9.0)	1.09	4.16	1.02
				−11.5			(0.25 mg/kg)							
							Alteplase							
							(0.9 mg/kg)							
TASTE	Australia, Belgium, Canada, Finland, New Zealand, Spain, Taiwan, and the UK	RCT	680	73.1	202 (60%)	218 (64%)	TNK	44 (13%)/35 (10%)	—	7 (4‐11)	7 (5‐10)	1.47	14.47	15.65
2024 (Parsons et al., 2024)				−13			(0.25 mg/kg)							
							Alteplase (0.90 mg/kg)							
ATTEST‐2 2024 (Muir et al. 2024)	UK	RCT	1777	70.4 (12.96)	533 (60%)	527 (59%)	TNK	94 (11%)/103 (12%)	180 (20%) / 170 (19%)	7 (5–13)	7 (5–12)	—	—	—
							(0.25 mg/kg)							
							Alteplase							
							(0.9 mg/kg)							

*Note*: * Mean ± SD

Abbreviations: ICA, internal carotid artery; IQR: interquartile range (twenty‐fifth to seventy‐fifth percentile); M1 and M2, segments of the MCA; MCA, middle cerebral artery; Med, Median; NIHSS, National Institutes of Health Stroke Scale; RCT, randomized controlled trial; SD, standard deviation; TNK, Tenecteplase.

### 3.3 Risk of Bias and GRADE Assessment

The Cochrane risk of bias (RoB 2.0) tool was used to evaluate the quality of RCTs (Sterne et al. 2019). Nine RCTs (Meng et al. 2024; Muir et al. 2024; Logallo et al. 2017; Wang et al. 2023; Menon et al. 2022; Li et al. 2022; Campbell et al. 2018; Parsons et al. 2012; Haley et al. 2010) showed a low risk of bias, with the remaining studies (Kvistad et al. 2022; Parsons et al. 2024; Bivard et al. 2022; Huang et al. 2015) demonstrating an uncertain risk of bias. Details of the quality assessment of RCTs were given in Supplementary Figure S1. To evaluate the certainty of evidence, the GRADE assessment of the outcomes was performed by GRADEpro GDT (Guyatt et al. 2011). The GRADE summary of findings table is provided as Table 2.

**TABLE 2:** GRADE assessment.

Tenecteplase compared to Alteplase for Acute Ischemic Stroke					
Patient or population: Acute ischemic stroke Intervention: Tenecteplase Comparison: Alteplase					
Outcomes	Anticipated absolute effects (95% CI)		Relative effect (95% CI)	№ of participants (studies)	Certainty of the evidence (GRADE)
	Risk with Alteplase	Risk with Tenecteplase			
Excellent functional outcome (mRS 0‐1) at 90 days	521 per 1000	**542 per 1000** (521 to 563)	**RR 1.04** (1.00 to 1.08)	8893 (13 RCTs)	⨁⨁⨁⨁ High
Mortality at 90 days	82 per 1000	**82 per 1,000** (68 to 98)	**RR 0.99** (0.83 to 1.19)	8985 (13 RCTs)	⨁⨁⨁◯ Moderate^a^
Good functional outcome (mRS 0–2) at 90 days	684 per 1000	**691 per 1000** (664 to 725)	**RR 1.01** (0.97 to 1.06)	8781 (12 RCTs)	⨁⨁⨁⨁ High
Poor functional outcome (mRS 5‐6) at 90 days	117 per 1000	**115 per 1000** (96 to 136)	**RR 0.98** (0.82 to 1.16)	7230 (12 RCTs)	⨁⨁⨁◯ Moderate^a^
Symptomatic ICH	21 per 1,000	**24 per 1000** (18 to 32)	**RR 1.16** (0.88 to 1.53)	8931 (12 RCTs)	⨁⨁⨁◯ Moderate^a^
Major neurological improvement (within 72 h)	445 per 1000	**459 per 1000** (423 to 503)	**RR 1.03** (0.95 to 1.13)	5652 (9 RCTs)	⨁⨁⨁◯ Moderate^a^

^a^ 95% CI crossing the 1;

Abbreviations: **CI**, confidence interval; **ICH**, intracranial hemorrhage; **RR**, risk ratio.

### 3.4 Outcomes

#### 3.4.1 Excellent Functional Outcome at 90 Days

**FIGURE 2:**
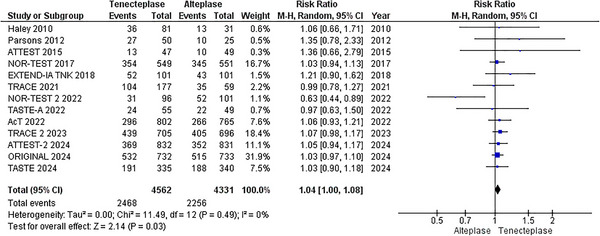
Excellent Functional Outcome (mRS 0–1) at 90 days.

A pairwise analysis of thirteen RCTs (Meng et al. 2024; Muir et al. 2024; Logallo et al. 2017; Kvistad et al. 2022; Parsons et al. 2024; Wang et al. 2023; Menon et al. 2022; Bivard et al. 2022; Li et al. 2022; Campbell et al. 2018; Huang et al. 2015; Parsons et al. 2012; Haley et al. 2010), with a total of 8893 patients (4562 TNK versus 4331 alteplase), revealed that patients receiving TNK had a statistically significant increase in excellent functional outcome at 90 days compared to alteplase (RR = 1.04; 95% CI: [1.00, 1.08]; p = 0.03; I^2^ = 0%), Figure 2. On network analysis, TNK 0.25 mg/kg showed significant improvement in excellent functional outcome compared to alteplase 0.9 mg/kg (RR = 1.05, 95% CI: [1.01, 1.10]; p < 0.01), Figures 6A and 7A. According to the P‐scores, TNK 0.25 mg/kg was ranked as the best (P‐score = 0.86), and TNK 0.1 mg/kg as the worst (P‐score = 0.16).

#### 3.4.2 Good Functional Outcome at 90 Days

**FIGURE 3:**
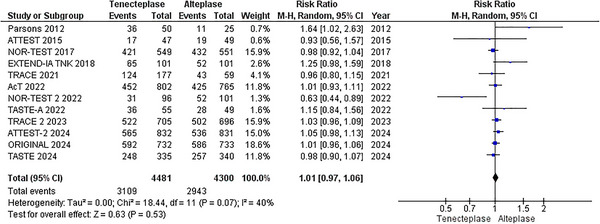
Good functional Outcome (mRS 0–2) at 90 days.

A pooled analysis of 12 RCTs (Meng et al. 2024; Muir et al. 2024; Logallo et al. 2017; Kvistad et al. 2022; Parsons et al. 2024; Wang et al. 2023; Menon et al. 2022; Bivard et al. 2022; Li et al. 2022; Campbell et al. 2018; Huang et al. 2015; Parsons et al. 2012), with 8,781 patients (4481 receiving TNK versus 4300 receiving alteplase), showed no statistically significant difference in good functional outcome among stroke patients receiving TNK versus alteplase (RR = 1.01; 95% CI: [0.97, 1.06]; p = 0.53; I^2^ = 40%), as shown in Figure 3. In network analysis, good functional outcome was significantly improved by TNK 0.32 mg/kg (RR = 1.30, 95% CI: [1.15, 1.48]; p < 0.01) compared to alteplase, Figures 6B and 7B. According to the P‐scores, TNK 0.32 was ranked as the most effective treatment (P‐score = 1.00), and TNK 0.1 mg/kg as the least effective (P‐score = 0.11).

#### 3.4.3 Major Neurological Improvement Within 72 H

**FIGURE 4:**
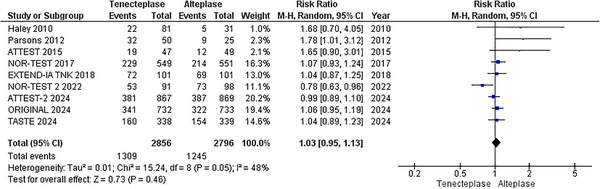
Major neurological Improvement within 72 h.

A pooled analysis of nine RCTs (Meng et al. 2024; Muir et al. 2024; Logallo et al. 2017; Kvistad et al. 2022; Parsons et al. 2024; Campbell et al. 2018; Huang et al. 2015; Parsons et al. 2012; Haley et al. 2010), with a total of 5652 patients (2856 TNK versus 2796 alteplase), revealed an increased rate of major neurological improvement with TNK compared to alteplase. However, these findings were not statistically significant (RR = 1.03; 95% CI: [0.95, 1.13]; p = 0.46; I^2^ = 48%), Figure 4. The network meta‐analysis showed no significant difference between various thrombolytic doses (Figures 6C and 7C). According to the P‐scores, TNK, 0.25 mg/kg, was ranked as the best treatment (P‐scores = 0.93).

#### 3.4.4 Mortality at 90 Days

**FIGURE 5:**
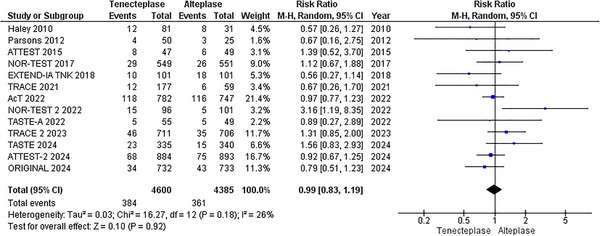
All‐cause mortality forest plot.

**FIGURE 6:**
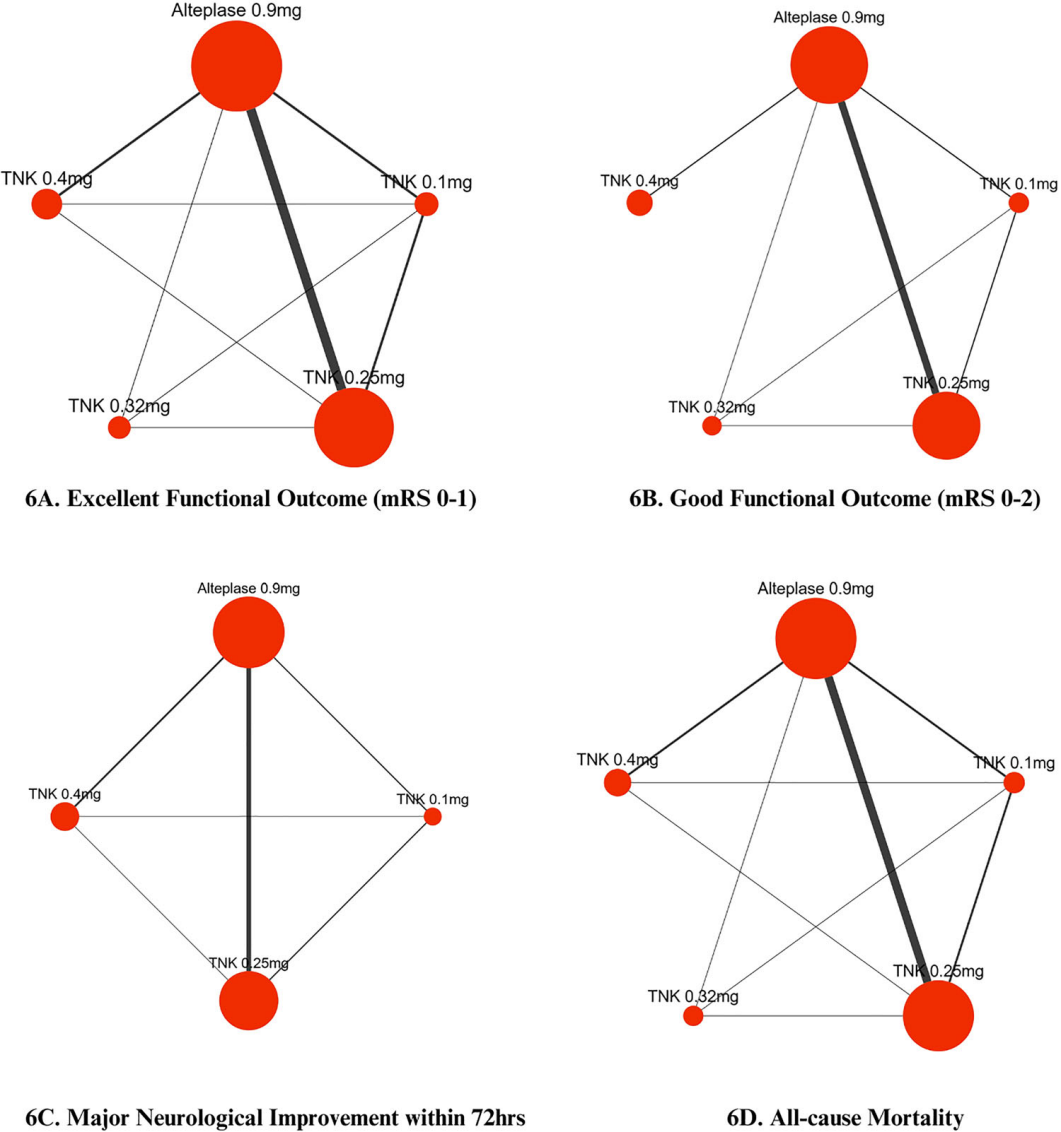
Network evidence plots for **(A)** Excellent functional Outcome (mRS 0–1), **(B)** Good functional Outcome (mRS 0–2), **(C)** Major neurological Improvement within 72 h and **(D)** All‐cause mortality.

**FIGURE 7:**
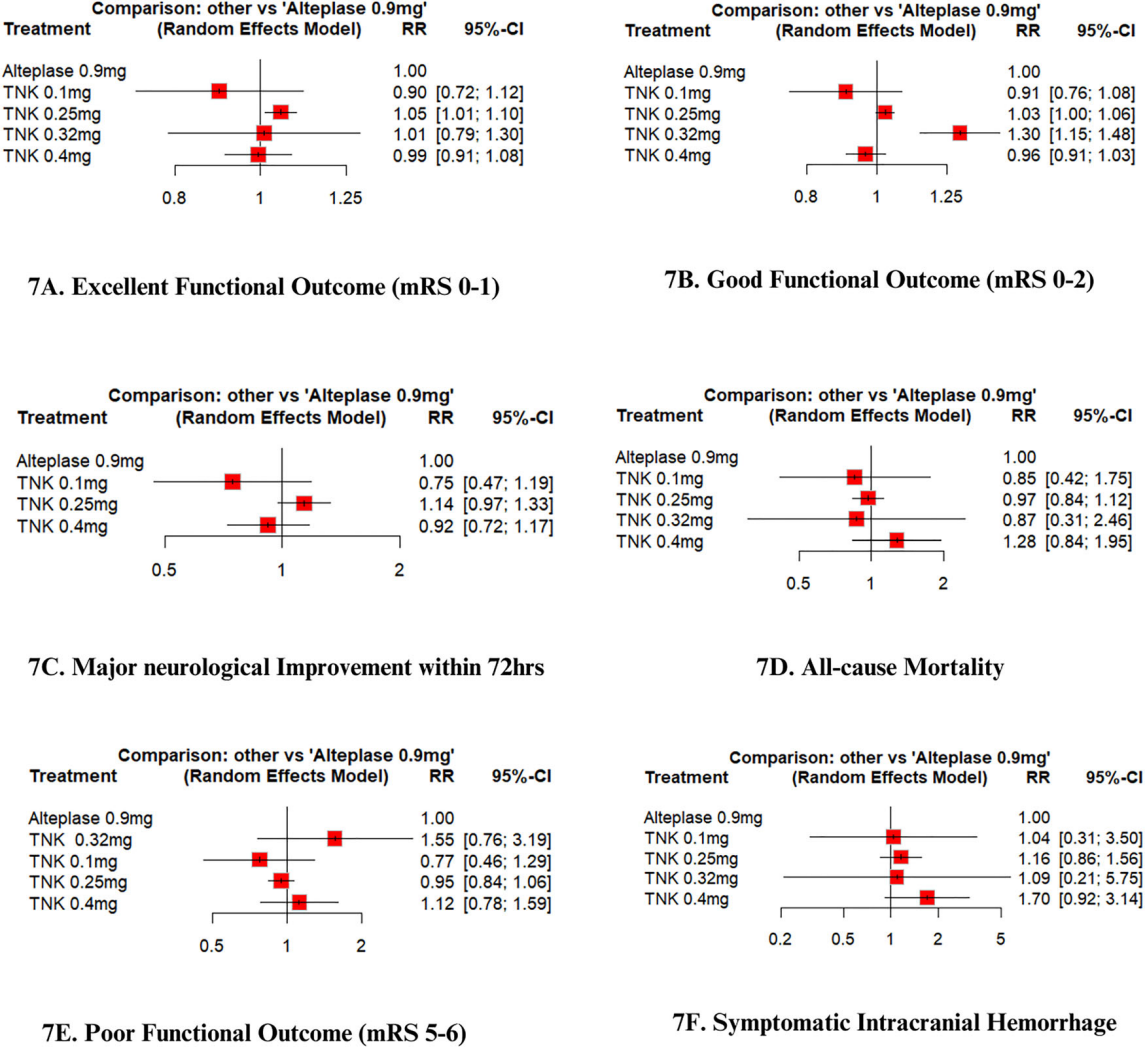
Network forest plots **(A)** Excellent functional outcome (mRS 0–1), **(B)** Good functional outcome (mRS 0‐2), **(C)** Major neurological improvement within 72 h **(D)** All‐cause mortality, **(E)** Poor functional outcome (mRS 5–6) and **(F)** Symptomatic intracranial hemorrhage.

Thirteen RCTs reported the outcome of mortality (Meng et al. 2024; Muir et al. 2024; Logallo et al. 2017; Kvistad et al. 2022; Parsons et al. 2024; Wang et al. 2023; Menon et al. 2022; Bivard et al. 2022; Li et al. 2022; Campbell et al. 2018; Huang et al. 2015; Parsons et al. 2012; Haley et al. 2010), comprising 8985 patients (4600 TNK versus 4385 alteplase). The overall pooled analysis revealed that TNK is comparable to alteplase regarding this outcome, with a pooled RR of 0.99 (95% CI: [0.83, 1.19]; p = 0.92; I^2^ = 26%), Figure 5. On network analysis, various thrombolytic doses showed no significant difference (Figures 6D and 7D). According to P‐scores, TNK 0.1 mg/kg was ranked as the best (P‐score = 0.66), and TNK 0.4 mg/kg as the worst (P‐score = 0.16) for the mortality outcome.

#### 3.4.5 Poor Functional Outcome at 90 Days

Twelve RCTs (Meng et al. 2024; Logallo et al. 2017; Kvistad et al. 2022; Parsons et al. 2024; Wang et al. 2023; Menon et al. 2022; Bivard et al. 2022; Li et al. 2022; Campbell et al. 2018; Huang et al. 2015; Parsons et al. 2012; Haley et al. 2010) reported poor functional outcome (mRS 5–6) at 90 days among 7230 patients (3730 TNK versus 3500 alteplase). The pairwise analysis yielded comparable results regarding this outcome between the two groups (RR = 0.98; 95% CI: [0.82, 1.16]; p = 0.81; I^2^ = 30%), as shown in Supplementary Figure S2. The network meta‐analysis showed no significant difference between various thrombolytic dosing regimens (Figure 7E). According to the P‐scores, TNK 0.1 mg/kg was ranked as the best option (P‐score = 0.86), and TNK 0.32 mg/kg as the worst (P‐score = 0.11) for poor functional outcome.

### 3.5 Symptomatic Intracranial Hemorrhage

Twelve RCTs reported the event of sICH (Meng et al. 2024; Muir et al. 2024; Logallo et al. 2017; Kvistad et al. 2022; Parsons et al. 2024; Wang et al. 2023; Menon et al. 2022; Li et al. 2022; Campbell et al. 2018; Huang et al. 2015; Parsons et al. 2012; Haley et al. 2010), comprising 8931 patients (4574 TNK versus 4357 alteplase). Pairwise analysis of these studies revealed that TNK and alteplase are comparable regarding symptomatic ICH (RR = 1.16; 95% CI: [0.88, 1.53]; p = 0.28; I^2^ = 0%), Supplementary Figure S3. The network analysis showed no significant difference between the different thrombolytic regimens (Figure 7F). The P‐scores ranked alteplase 0.9 mg/kg as the best (P‐score = 0.71) and TNK 0.4 mg/kg as the worst (P‐score = 0.17) for sICH.

### 3.6 Sensitivity Analysis

We performed a leave‐one‐out sensitivity analysis for the pooled estimates where cumulative heterogeneity exceeded 40%. On removing the NOR‐TEST 2 2022 study (Kvistad et al. 2022), the heterogeneity decreases from 40% to 11% in the good functional outcome, as shown in Supplementary Figure S4. Similarly, by removing the same study (Kvistad et al. 2022), the heterogeneity of major neurological improvement within 72 h decreased from 48% to 14%, Supplementary Figure S5.

### 3.7 Publication Bias

For outcomes pooling more than 10 RCTs, we analyzed the publication bias visually through funnel plots and statistically through Egger's regression test. In Egger's regression test, a *p*‐value of less than 0.05 was deemed statistically significant. No significant publication bias was found. Funnel plots of these outcomes are depicted in Supplementary Figures S6–11.

### 3.8 GRADE Assessment

When assessing the certainty of evidence through the GRADE assessment, the excellent and good functional outcomes demonstrated high certainty of evidence, while the remaining endpoints, including mortality, poor functional outcome, major neurological improvement, and sICH, showed moderate certainty of evidence.

### 3.9 Inconsistency and League Tables

The inconsistency between the direct and indirect evidence was determined using the node‐splitting analysis. There was no significant inconsistency in any outcome. The details are presented in Supplementary Tables S2–7. The network estimates for the different comparisons between the various thrombolytic regimens are provided as league tables and Supplementary Figures S12–17.

## 4 Discussion

This pairwise and network meta‐analysis, involving 13 RCTs and 9044 patients, thoroughly assesses the effectiveness and safety of different TNK doses compared with alteplase for treating AIS. The findings of the pairwise meta‐analysis show that at 90 days, TNK demonstrated a statistically significant increase in excellent functional outcome. However, both treatments are associated with comparable results in terms of good functional outcome and poor functional outcome. Regarding the risk of sICH, no significant difference exists between the two treatments. Furthermore, TNK and alteplase are equally effective in achieving a major neurological improvement within 72 h. The two drugs are also similar in terms of mortality at 90 days.

This quantitative analysis established that TNK is more likely than alteplase to achieve excellent functional outcomes at 90 days in individuals who suffered AIS. This contributes to the growing evidence indicating that TNK offers a significant benefit over alteplase in achieving excellent functional outcomes. This observation aligns with a meta‐analysis by Shen et al. (2023). On the contrary, this contradicts the findings from the meta‐analysis by Huang et al. (2024) and Kheiri et al. (2018) that found no clear advantage of one thrombolytic agent over the other. This difference highlights the development of knowledge regarding the effectiveness of TNK. The network meta‐analysis by Rehman et al. showed that the TNK 0.25 mg/kg regimen ranked as the most effective for excellent functional outcome, which supports the current evidence (Rehman et al. 2023). Our results offer enhanced reliability compared to previous studies, as we exclusively included randomized controlled trials, thereby minimizing potential biases associated with non‐randomized designs. Additionally, adding the recent high‐quality RCTs like ORIGINAL and ATTEST‐2 increased the statistical power. Furthermore, we used a frequentist network meta‐analysis for dose‐specific comparisons and treatment rankings.

Our meta‐analysis corroborated the findings of previous studies that have shown no significant difference in achieving good functional outcome and poor functional outcome at 90 days between the two arms. This showed that both treatments are equally effective in enhancing functional recovery in AIS patients (Huang et al. 2024; Potla and Ganti 2022; Rehman et al. 2023). In the network analysis of various doses, TNK 0.32 mg/kg was ranked as the best treatment regarding good functional outcome, and TNK 0.1 mg/kg as the worst. The pooled analysis on major neurological improvement showed no statistically significant difference between the two thrombolytic agents. This finding aligns with the various meta‐analyses performed previously, such as the one by Shen et al. (Shen et al. 2023), which reported no significant difference in early neurological improvement between TNK and alteplase. Meanwhile, the relative ranking of different treatment doses showed TNK 0.25 as the best‐ranked treatment. This supports the findings of other studies (Rehman et al. 2023). No statistically significant difference in the incidence of sICH was found between TNK and alteplase. This finding is consistent with the findings from other meta‐analysis studies (Ezzeldin et al. 2023; Warach et al. 2020; Katsanos et al. 2022), thus reinforcing the safety profile of TNK. In network analysis for sICH, alteplase 0.9 mg/kg was ranked as the most effective regimen, and TNK 0.4 mg/kg was ranked as the least effective. This indicates an increasing risk of sICH with higher TNK doses (Rehman et al. 2023).

The pairwise analysis for 90‐day mortality showed no statistically significant difference regarding this outcome between the two thrombolytic agents, and these findings were consistent with the studies done previously (Xu et al. 2018; Rose et al. 2023; Kobeissi et al. 2023). Nevertheless, the meta‐analysis conducted by Emberson et al. (2014) suggested that to gain a comprehensive understanding of the impact of alteplase on mortality, it is necessary to collect longer‐term follow‐up data. Upon comparative analysis of various doses, TNK 0.1 mg/kg was ranked as the most effective and TNK 0.4 mg/kg as the least effective regimen for all‐cause mortality.

TNK is referred to as a genetically engineered form of alteplase with a longer half‐life of 24 min, compared to 4–5 min for alteplase, and a high affinity towards fibrin, which enhances its thrombolytic activity (Shen et al. [Link], 2023.). This makes the drug suitable for administration with a rapid bolus, which enables a short action time and may promote improved recanalization of occluded arteries, a benefit in the treatment of AIS (Yogendrakumar et al. 2023). The early recanalization of vessels and neurological improvement following TNK administration may be attributed to its faster plasmin generation, which facilitates the restoration of blood flow to ischemic brain areas more quickly (Yogendrakumar et al. 2023). Early recanalization at this level results in improved functional outcomes, as swiftly restoring the blood supply reduces ischemic injury and enhances recovery (Haley et al. 2010). The increased fibrin specificity of TNK may also have a significant impact on lowering bleeding risks, as it selectively binds to fibrin‐bound plasminogen and reduces the possibility of systemic effects (Shen et al. [Link], 2023.). The pharmacological properties of TNK, including its rapid time of action, higher fibrin specificity, and ease of administration, make it a more effective option than alteplase for treating AIS. The latter is anticipated to offer functional recovery and safety advantages. However, multiple different doses of TNK still leave room for more research to determine the optimal dosing regimen in patients with AIS.

A recent study conducted by Ketabforoush et al. emphasizes the practical benefits of TNK, including its single‐bolus administration, extended half‐life, and enhanced fibrin specificity, all of which could aid in early vessel recanalization and boost workflow efficiency (Haj Mohamad Ebrahim Ketabforoush et al. 2024). These traits are particularly beneficial in large vessel occlusion (LVO) strokes, as TNK is associated with better procedural outcomes and faster reperfusion. These findings reinforce our results, highlighting the functional efficacy of TNK 0.25 mg/kg.

This study has its distinctive merits. This study represents the most recent and extensive meta‐analysis of randomized data on the efficacy of TNK compared to alteplase in the context of AIS therapy, based on a comprehensive sample of 9,044 patients synthesized from a total of 13 RCTs, which enhances the generalizability and reliability of the findings. A detailed network meta‐analysis was also conducted to compare doses of two thrombolytic agents and determine the optimal dosing regimen. In addition, the analysis reported several critical clinical endpoints, including survival rates and excellent functional status, with minimal heterogeneity in several studies, which strengthened the level of certainty about the findings.

However, there are a few limitations; most of the included studies analyzed the endpoints up to a 90‐day time window, which restricts insight regarding long‐term recovery, late adverse effects, and sustained treatment benefits. Extended follow‐up is necessary for assessing cognitive function, stroke recurrence, and quality of life, which are key factors for developing guidelines. Future RCTs should focus on long‐term outcome data to improve clinical decision‐making. Similarly, another limitation of our study is the lack of subgroup analysis on patients who received TNK before MT, as most included studies did not report relevant outcomes for this group. Future research should focus on assessing thrombolytic therapy prior to MT to provide clearer insights. Additionally, we limited the screening to studies that were published in English, which may have introduced selection bias. Overall, this meta‐analysis makes a solid claim that TNK is more effective than alteplase in patients with an AIS, and the preferred doses of TNK are 0.25 mg/kg and 0.32 mg/kg. However, further studies in the form of high‐quality RCTs are warranted to address the claim and the limitations stated.

## 5 Conclusion

This study, which pools data from 13 RCTs involving over 9,000 patients, presents the most comprehensive and up‐to‐date comparison of TNK doses with alteplase for AIS. By utilizing both pairwise and frequentist network meta‐analyses approaches, our study showed a statistically significant improvement in functional outcomes in the TNK arm, especially with 0.25 mg/kg and 0.32 mg/kg dosages, whereas the safety endpoints, including sICH and mortality, were comparable between the TNK and alteplase. These findings will assist clinicians in selecting the most effective IV thrombolytic, along with the optimal dose for treating the AIS.

## Author Contributions

**Muhammad Hassan Waseem**: conceptualization, writing – original draft, writing – review and editing, and supervision. **Zain ul Abideen**: writing – original draft, writing – review and editing, and data curation. **Muhammad Haris Khan**: writing – original draft. **Muhammad Fawad Tahir**: writing – original draft. **Marium Khan**: writing – review and editing. **Hafsa Arshad Azam Raja**: writing—review and editing. **Ameer Haider Cheema**: writing – original draft. **Sania Aimen**: writing – review and editing. **Muhammad Arslan Tariq**: writing – review and editing. **Javed Iqbal**: writing – review and editing.

## Ethics Statement

Not Applicable.

## Consent

The authors have nothing to report.

## Conflicts of Interest

The authors declare that they have no conflict of interest

## Peer Review

The peer review history for this article is available at https://publons.com/publon/10.1002/brb3.70756.

## Supporting information




**Supplementary Materials**: brb370756‐sup‐0001‐SuppMat.docx

## Data Availability

Data will be made available upon reasonable request to the authors.
