# Supplementary material for: Comparative Efficacy and Safety of Different Tenecteplase Doses With Alteplase in Acute Ischemic Stroke: A Systematic Review With Pairwise and Network Meta‐Analysis to Determine the Optimal Dose
Source: Brain Behav. 2025 Aug 19;15(8):e70756. doi: 10.1002/brb3.70756 (PMC12362178; doi:10.1002/brb3.70756)
Supplement: Supplementary file 1 — Supplementary Materials: brb370756‐sup‐0001‐SuppMat.docx [file BRB3-15-e70756-s001.docx]

| **Section and Topic** | **Item #** | **Checklist item** | **Location where item is reported** |
| --- | --- | --- | --- |
| **TITLE** | | |  |
| Title | 1 | Identify the report as a systematic review. | Title page, manuscript |
| **ABSTRACT** | | |  |
| Abstract | 2 | See the PRISMA 2020 for Abstracts checklist. | Abstract |
| **INTRODUCTION** | | |  |
| Rationale | 3 | Describe the rationale for the review in the context of existing knowledge. | Last paragraph of intro |
| Objectives | L | Provide an explicit statement of the objective(s) or question(s) the review addresses. | Last paragraph of intro |
| **METHODS** | | |  |
| Eligibility criteria | 5 | Specify the inclusion and exclusion criteria for the review and how studies were grouped for the syntheses. | Methods second paragraph |
| Information sources | 6 | Specify all databases, registers, websites, organisations, reference lists and other sources searched or consulted to identify studies. Specify the date when each source was last searched or consulted. | Literature search |
| Search strategy | 7 | Present the full search strategies for all databases, registers and websites, including any filters and limits used. | Literature search |
| Selection process | 8 | Specify the methods used to decide whether a study met the inclusion criteria of the review, including how many reviewers screened each record and each report retrieved, whether they worked independently, and if applicable, details of automation tools used in the process. | Literature search and eligibility criteria |
| Data collection process | 9 | Specify the methods used to collect data from reports, including how many reviewers collected data from each report, whether they worked independently, any processes for obtaining or confirming data from study investigators, and if applicable, details of automation tools used in the process. | Data extraction |
| Data items | 10a | List and define all outcomes for which data were sought. Specify whether all results that were compatible with each outcome domain in each study were sought (e.g. for all measures, time points, analyses), and if not, the methods used to decide which results to collect. | Data extraction |
|  | 10b | List and define all other variables for which data were sought (e.g. participant and intervention characteristics, funding sources). Describe any assumptions made about any missing or unclear information. | Data extraction paragraph |
| Study risk of bias assessment | 11 | Specify the methods used to assess risk of bias in the included studies, including details of the tool(s) used, how many reviewers assessed each study and whether they worked independently, and if applicable, details of automation tools used in the process. | Quality assessment |
| Effect measures | 12 | Specify for each outcome the effect measure(s) (e.g. risk ratio, mean difference) used in the synthesis or presentation of results. | Statistical analysis |
| Synthesis methods | 13a |  |  |
|  | 13b | Describe any methods required to prepare the data for presentation or synthesis, such as handling of missing summary statistics, or data conversions. | Statistical anlaysis |
|  | 13c | Describe any methods used to tabulate or visually display results of individual studies and syntheses. | Statistical analysis |
|  | 13d | Describe any methods used to synthesize results and provide a rationale for the choice(s). If meta-analysis was performed, describe the model(s), method(s) to identify the presence and extent of statistical heterogeneity, and software package(s) used. | Statistical analysis |
|  | 13e | Describe any methods used to explore possible causes of heterogeneity among study results (e.g. subgroup analysis, meta-regression). | Statistical analysis |
|  | 13f | Describe any sensitivity analyses conducted to assess robustness of the synthesized results. | Statistical analysis |
| Reporting bias assessment | 14 | Describe any methods used to assess risk of bias due to missing results in a synthesis (arising from reporting biases). |  |
| Certainty assessment | 15 | Describe any methods used to assess certainty (or confidence) in the body of evidence for an outcome. | Quality assessment, |
| **RESULTS** | | |  |
| Study selection | 16a | Describe the results of the search and selection process, from the number of records identified in the search to the number of studies included in the review, ideally using a flow diagram. | Search results |
|  | 16b | Cite studies that might appear to meet the inclusion criteria, but which were excluded, and explain why they were excluded. | Exclusion criteria |
| Study characteristics | 17 | Cite each included study and present its characteristics. | Table 1 |
| Risk of bias in studies | 18 | Present assessments of risk of bias for each included study. | Risk of bias paragraph |
| Results of individual studies | 19 | For all outcomes, present, for each study: (a) summary statistics for each group (where appropriate) and (b) an effect estimate and its precision (e.g. confidence/credible interval), ideally using structured tables or plots. | Results |
| Results of syntheses | 20a | For each synthesis, briefly summarise the characteristics and risk of bias among contributing studies. | Results |
|  | 20b | Present results of all statistical syntheses conducted. If meta-analysis was done, present for each the summary estimate and its precision (e.g. confidence/credible interval) and measures of statistical heterogeneity. If comparing groups, describe the direction of the effect. | Results |
|  | 20c | Present results of all investigations of possible causes of heterogeneity among study results. | Results |
|  | 20d | Present results of all sensitivity analyses conducted to assess the robustness of the synthesized results. | Results |
| Reporting biases | 21 | Present assessments of risk of bias due to missing results (arising from reporting biases) for each synthesis assessed. | Results |
| Certainty of evidence | 22 | Present assessments of certainty (or confidence) in the body of evidence for each outcome assessed. | GRADE |
| **DISCUSSION** | | |  |
|  | 23a | Provide a general interpretation of the results in the context of other evidence. | Discussion |
|  | 23b | Discuss any limitations of the evidence included in the review. | Limitations paragraph |
|  | 23c | Discuss any limitations of the review processes used. |  |
|  | 23d | Discuss implications of the results for practice, policy, and future research. |  |
| **OTHER INFORMATION** | | |  |
| Registration and protocol | 24a | Provide registration information for the review, including register name and registration number, or state that the review was not registered. | PROSPERO |
|  | 24b | Indicate where the review protocol can be accessed, or state that a protocol was not prepared. |  |
|  | 24c | Describe and explain any amendments to information provided at registration or in the protocol. |  |
| Support | 25 | Describe sources of financial or non-financial support for the review, and the role of the funders or sponsors in the review. | None |
| Competing interests | 26 | Declare any competing interests of review authors. | Statement |
| Availability of data, code and other materials | 27 | Report which of the following are publicly available and where they can be found: template data collection forms; data extracted from included studies; data used for all analyses; analytic code; any other materials used in the review. | Statement added at end |

*From:*  Page MJ, McKenzie JE, Bossuyt PM, Boutron I, Hoffmann TC, Mulrow CD, et al. The PRISMA 2020 statement: an updated guideline for reporting systematic reviews. BMJ 2021;372:n71. doi: 10.1136/bmj.n71. This work is licensed under CC BY 4.0. To view a copy of this license, visit <https://creativecommons.org/licenses/by/4.0/>

| **Supplementary Table 1:** Search Strategies Used in Different Databases | | |
| --- | --- | --- |
| **Database used** | **Search Strategy** | **Articles Retrieved** |
| PubMed | ("Tissue Plasminogen Activator"[Mesh] OR Plasminogen Activator, Tissue OR Tissue Activator D-44 OR Tissue Activator D 44 OR Plasminogen Activator, Tissue-Type OR Plasminogen Activator, Tissue Type OR Tissue-Type Plasminogen Activator OR Tissue Type Plasminogen Activator OR TTPA OR T-Plasminogen Activator OR T Plasminogen Activator OR Alteplase OR Tisokinase OR Actilyse OR Lysatec rt-PA OR Lysatec rtPA OR Lysatec rt PA OR Activase) AND ("Tenecteplase"[Mesh] OR Metalyse OR TNKase) AND ("Ischemic Stroke"[Mesh] OR Ischemic Strokes OR Stroke, Ischemic OR Ischaemic Stroke OR Ischaemic Strokes OR Stroke, Ischaemic OR Acute Ischemic Stroke OR Acute Ischemic Strokes OR Ischemic Stroke, Acute OR Stroke, Acute Ischemic OR Cryptogenic Ischemic Stroke OR Cryptogenic Ischemic Strokes OR Ischemic Stroke, Cryptogenic OR Stroke, Cryptogenic Ischemic OR Cryptogenic Embolism Stroke OR Cryptogenic Embolism Strokes OR Embolism Stroke, Cryptogenic OR Stroke, Cryptogenic Embolism OR Cryptogenic Stroke OR Cryptogenic Strokes OR Stroke, Cryptogenic OR Wake-up Stroke OR Stroke, Wake-up OR Wake up Stroke OR Wake-up Strokes) | 376 |
| Cochrane Library | ("Tissue Plasminogen Activator"[Mesh] OR Alteplase OR Tisokinase OR Actilyse ORActivase) AND ("Tenecteplase"[Mesh] OR Metalyse OR TNKase) AND ("Ischemic Stroke"[Mesh] OR Ischemic Strokes OR Acute Ischemic Stroke) | 227 |
| ScienceDirect | ("Tissue Plasminogen Activator" OR Alteplase OR Tisokinase OR Activase) AND ("Tenecteplase" OR Metalyse OR TNKase) AND ("Ischemic Stroke" OR Acute Ischemic Stroke) | 1,064 |


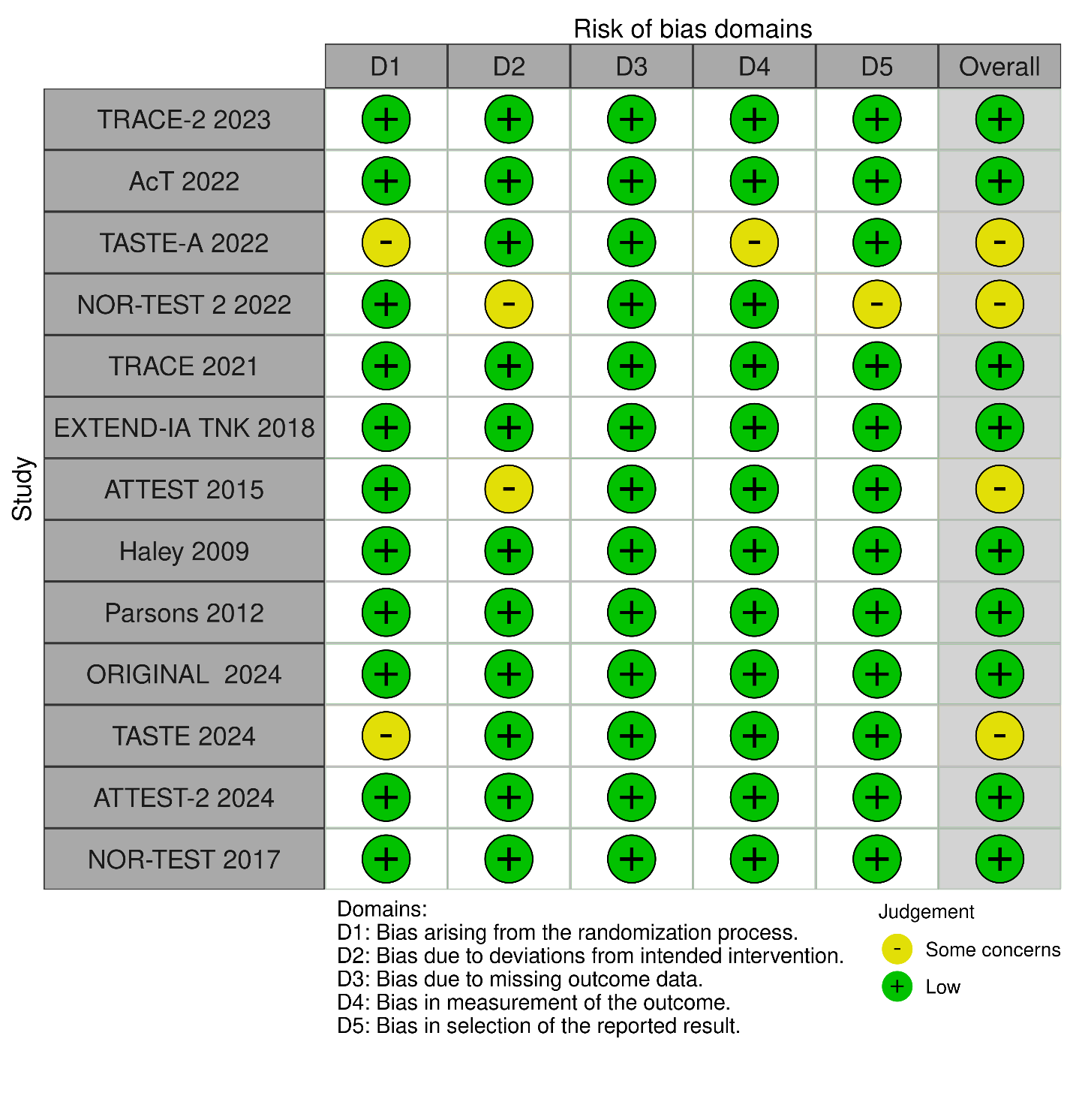


**Supplementary Figure S1:** Quality assessment of RCTs by the Cochrane RoB2.0 tool


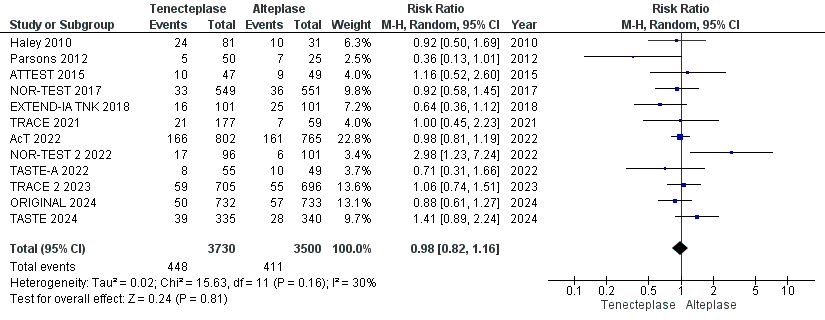
**Supplementary Figure S2:** Poor Functional Outcome (mRS 5-6) at 90 days


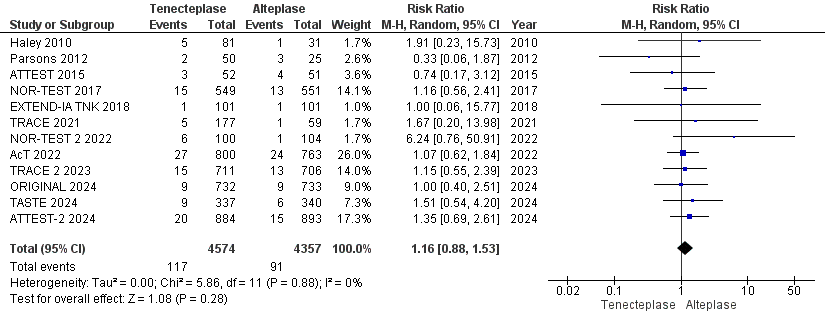


**Supplementary Figure S3:** Symptomatic Intracranial Hemorrhage

**
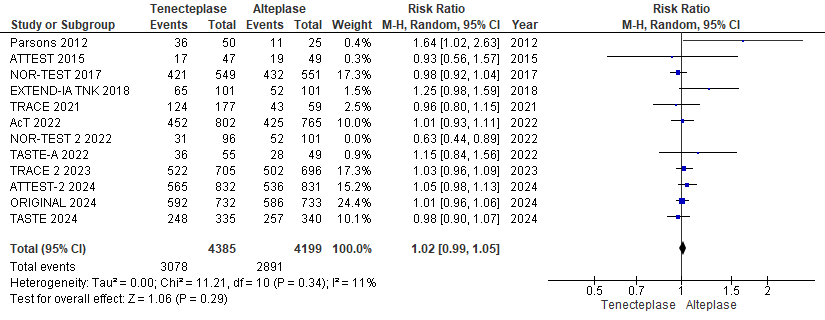
**

**Supplementary Figure S4:** Leave-one-out sensitivity analysis plot of Good functional outcome (mRS 0-2) at 90 days

**
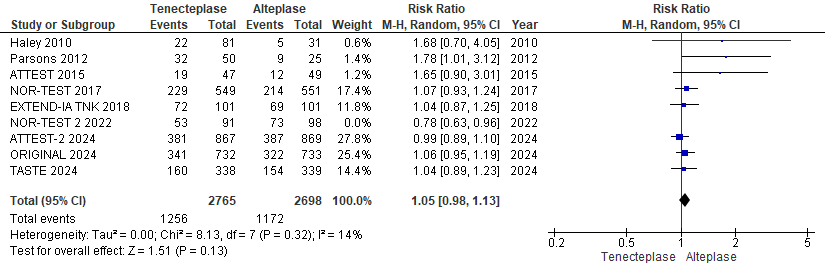
**

**Supplementary Figure S5:** Leave-one-out sensitivity analysis plot of Major neurological improvement within 72 hours

**
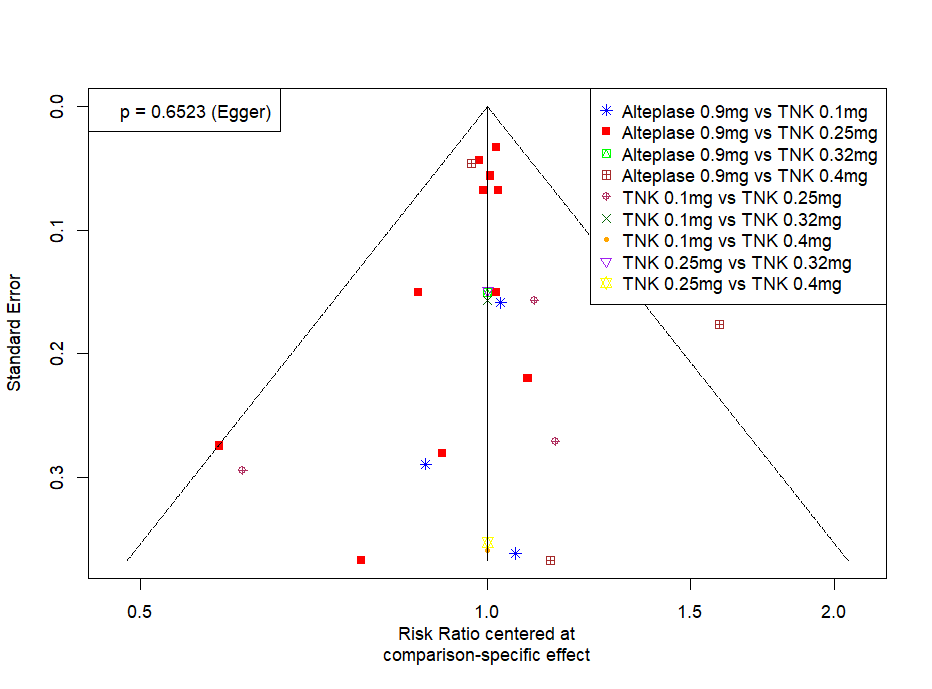
**

**Supplementary Figure S6: Funnel plot for excellent functional outcome**

**
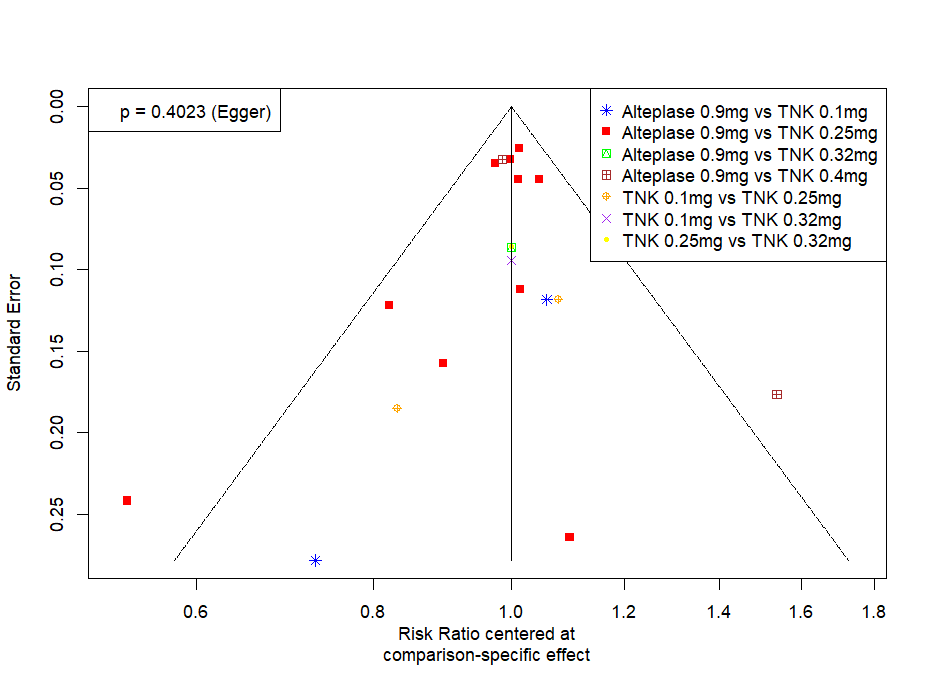
**

**Supplementary Figure S7: Funnel plot for good functional outcome**

**
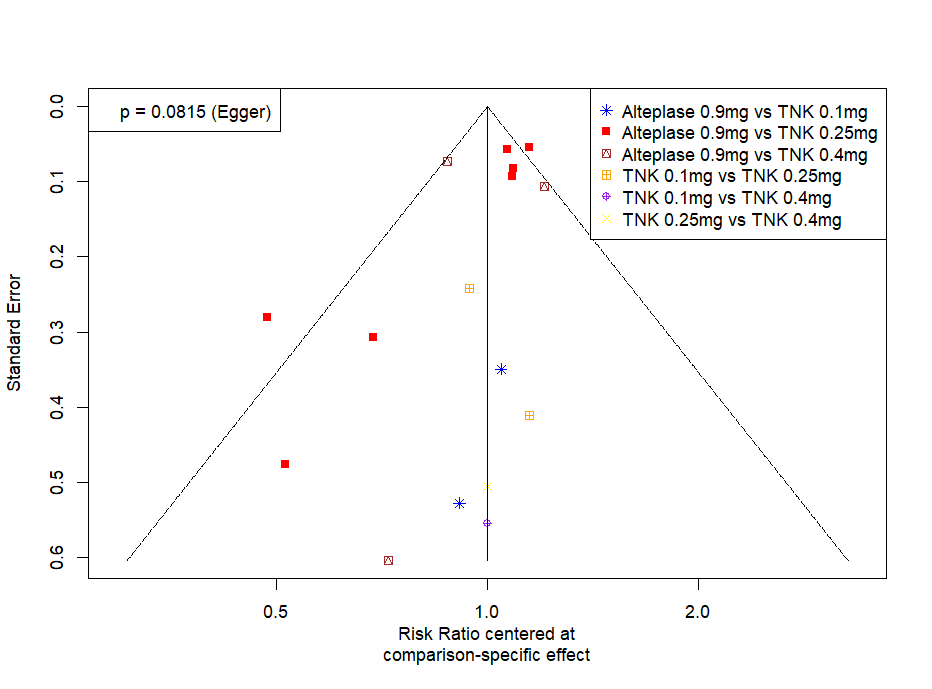
**

**Supplementary Figure S8: Funnel plot for Major Neurological Improvement within 72hrs**

**
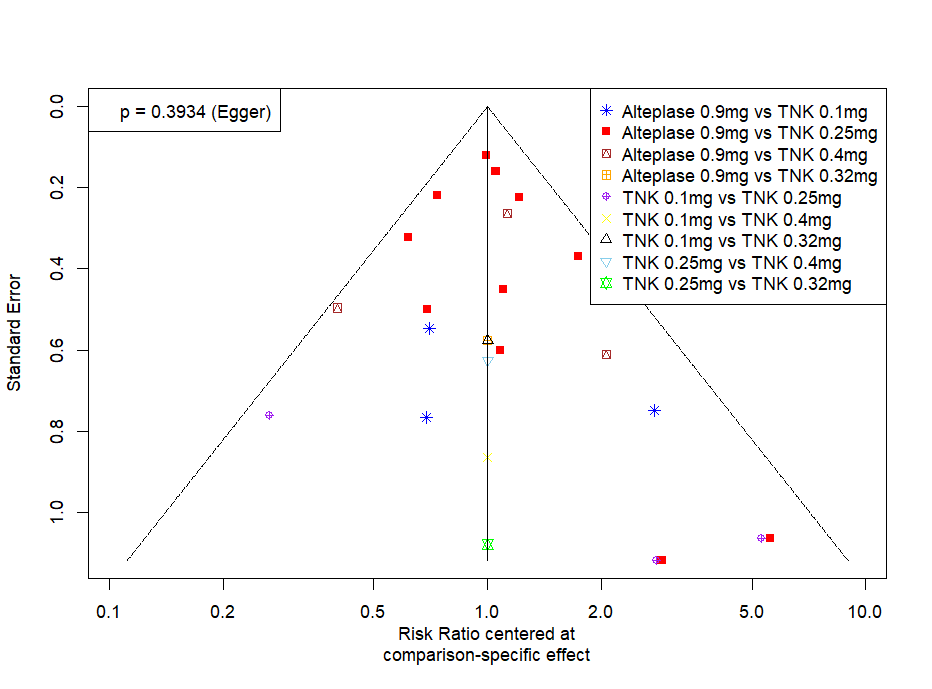
**

**Supplementary Figure S9: Funnel plot for mortality**

**
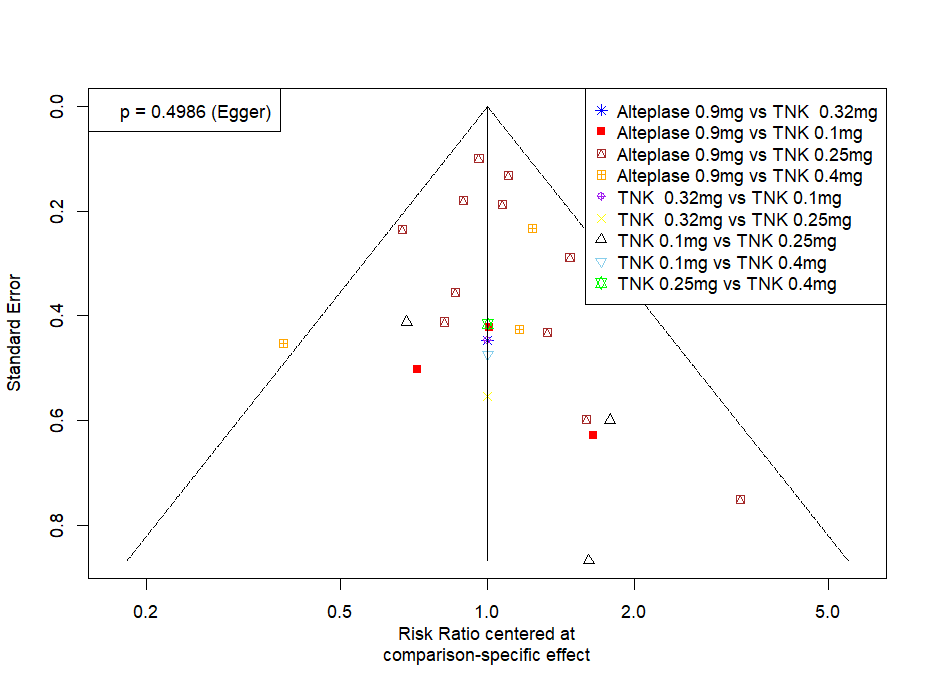
**

**Supplementary Figure S10: Poor Functional outcome (mRS 5-6)**

**
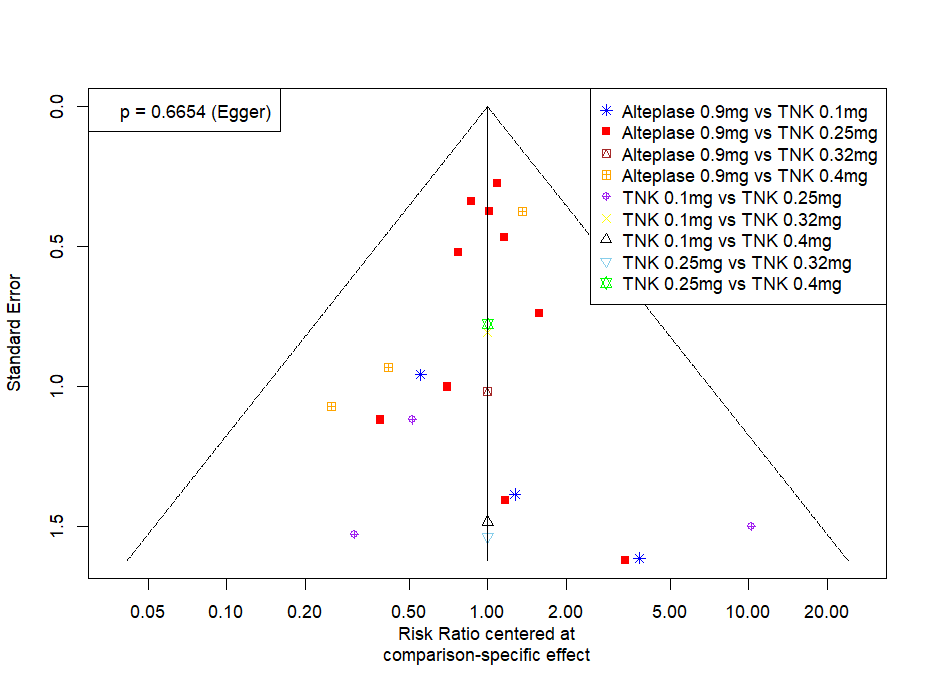
**

**Supplementary Figure S11: Funnel plot for Symptomatic Intracranial Hemorrhage**

| **Supplementary Table S2: Node-analysis for inconsistency in excellent functional outcome** | | | |
| --- | --- | --- | --- |
| **Comparison** | **Direct** | **indirect** | **p-value** |
| **TNK 0.1mg vs Alteplase 0.9mg** | 0.9524 | 0.7695 | 0.4049 |
| **TNK 0.1mg vs TNK 0.25mg** | 0.8137 | 1.0155 | 0.442 |
| **TNK 0.1mg vs TNK 0.32mg** | 0.9167 | 0.7463 | 0.6271 |
| **TNK 0.1mg vs TNK 0.4mg** | 1.2258 | 0.885 | 0.3975 |
| **TNK 0.25mg vs Alteplase 0.9mg** | 1.0583 | 1.9838 | 0.2293 |
| **TNK 0.25mg vs TNK 0.32mg** | 1.0234 | 1.1209 | 0.7594 |
| **TNK 0.25mg vs TNK 0.4mg** | 1.3134 | 1.0778 | 0.5829 |
| **TNK 0.32mg vs Alteplase 0.9mg** | 1.0114 | 1.0104 | 0.9972 |
| **TNK 0.4mg vs Alteplase 0.9mg** | 0.9812 | 0.5753 | 0.4386 |

| **Supplementary Table S3: Node-splitting analysis for inconsistency in Good functional outcome (mRS 0-2)** | | | |
| --- | --- | --- | --- |
| **Comparison** | **direct** | **indirect** | **p-value** |
| **TNK 0.1mg vs Alteplase 0.9mg** | 0.9987 | 0.7295 | 0.1382 |
| **TNK 0.1mg vs TNK 0.25mg** | 0.856 | 1.0014 | 0.5435 |
| **TNK 0.1mg vs TNK 0.32mg** | 0.7167 | 0.5852 | 0.4575 |
| **TNK 0.25mg vs Alteplase 0.9mg** | 1.0337 | 1.6315 | 0.4575 |
| **TNK 0.25mg vs TNK 0.32mg** | 0.7728 | 0.8205 | 0.6978 |
| **TNK 0.32mg vs Alteplase 0.9mg** | 1.3083 | 1.305 | 0.9873 |

| **Supplementary Figure S4: Major neurological Improvement within 72hrs** | | | |
| --- | --- | --- | --- |
| **Comparison** | **direct** | **indirect** | **p-value** |
| **TNK 0.1mg vs Alteplase 0.9mg** | 1.2762 | 0.3545 | 0.005 |
| **TNK 0.1mg vs TNK 0.25mg** | 0.5537 | 6.3595 | 0.0074 |
| **TNK 0.1mg vs TNK 0.4mg** | 1.0726 | 0.7192 | 0.5262 |
| **TNK 0.25mg vs Alteplase 0.9mg** | 1.1083 | 1.5564 | 0.6114 |
| **TNK 0.25mg vs TNK 0.4mg** | 1.6855 | 1.1768 | 0.5022 |
| **TNK 0.4mg vs Alteplase 0.9mg** | 0.9444 | 0.2752 | 0.1419 |

| **Supplementary Table S5: Node-splitting analysis for inconsistency in mortality outcome** | | | |
| --- | --- | --- | --- |
| **Comparison** | **direct** | **indirect** | **p-value** |
| **TNK 0.1mg vs Alteplase 0.9mg** | 0.6844 | 8.8002 | 0.0579 |
| **TNK 0.1mg vs TNK 0.25mg** | 1.0971 | 0.7136 | 0.5809 |
| **TNK 0.1mg vs TNK 0.32mg** | 1.2 | 0.2456 | 0.3447 |
| **TNK 0.1mg vs TNK 0.4mg** | 0.4086 | 0.7732 | 0.5351 |
| **TNK 0.25mg vs Alteplase 0.9mg** | 0.9556 | 9.9737 | 0.0777 |
| **TNK 0.25mg vs TNK 0.32mg** | 0.2105 | 2.0007 | 0.078 |
| **TNK 0.25mg vs TNK 0.4mg** | 1.4301 | 0.6492 | 0.2711 |
| **TNK 0.32mg vs Alteplase 0.9mg** | 0.8194 | 1.2708 | 0.7726 |
| **TNK 0.4mg vs Alteplase 0.9mg** | 1.2899 | 2.2457 | 0.7394 |

| **Supplementary Table S6: Poor functional Outcome (mRS 5-6)** | | | |
| --- | --- | --- | --- |
| **Comparison** | **direct** | **indirect** | **p-value** |
| **TNK 0.32mg vs Alteplase 0.9mg** | 1.5452 | 1.5819 | 0.9769 |
| **TNK 0.32mg vs TNK 0.1mg** | 1.5714 | 4.1353 | 0.2904 |
| **TNK 0.32mg vs TNK 0.25mg** | 2.6125 | 1.126 | 0.2723 |
| **TNK 0.1mg vs Alteplase 0.9mg** | 0.7051 | 1.2074 | 0.4473 |
| **TNK 0.1mg vs TNK 0.25mg** | 0.9402 | 0.6025 | 0.4519 |
| **TNK 0.1mg vs TNK 0.4mg** | 0.7151 | 0.6577 | 0.8967 |
| **TNK 0.25mg vs Alteplase 0.9mg** | 0.9379 | 1.4952 | 0.5702 |
| **TNK 0.25mg vs TNK 0.4mg** | 1.1237 | 0.7566 | 0.4206 |
| **TNK 0.4mg vs Alteplase 0.9mg** | 1.1606 | 0.7858 | 0.6515 |

| **Supplementary Table S7: Node splitting analysis for inconsistency in sICH** | | | |
| --- | --- | --- | --- |
| **Comparison** | **direct** | **Indirect** | **p-value** |
| **TNK 0.1mg vs Alteplase 0.9mg** | 1.2734 | 0.5343 | 0.5522 |
| **TNK 0.1mg vs TNK 0.25mg** | 0.6483 | 1.6257 | 0.4796 |
| **TNK 0.1mg vs TNK 0.32mg** | 1.4 | 0.0069 | 0.076 |
| **TNK 0.1mg vs TNK 0.4mg** | 0.0884 | 1.02 | 0.1429 |
| **TNK 0.25mg vs Alteplase 0.9mg** | 1.1669 | 0.732 | 0.6764 |
| **TNK 0.25mg vs TNK 0.32mg** | 0.2104 | 2.1592 | 0.2074 |
| **TNK 0.25mg vs TNK 0.4mg** | 0.4422 | 0.7538 | 0.5366 |
| **TNK 0.32mg vs Alteplase 0.9mg** | 1.6391 | 0.4334 | 0.4699 |
| **TNK 0.4mg vs Alteplase 0.9mg** | 1.5762 | 3.2872 | 0.4771 |

**
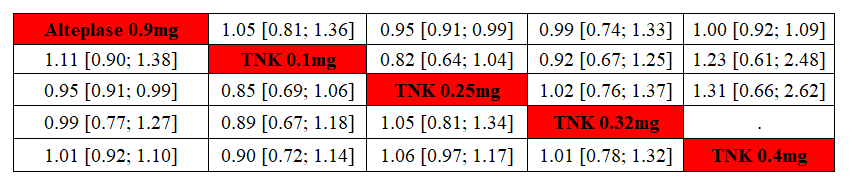
**

**Supplementary Figure S12: League table for excellent functional outcome**

**
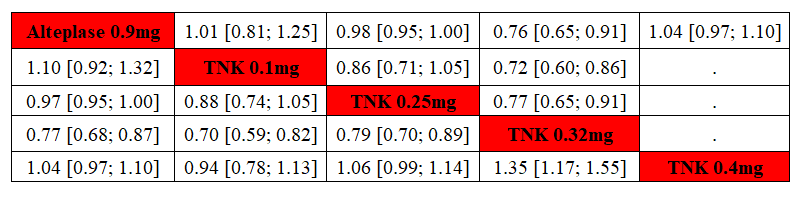
**

**Supplementary Figure S13: League Table for Good Functional Outcome (mRS 0-2)**

**
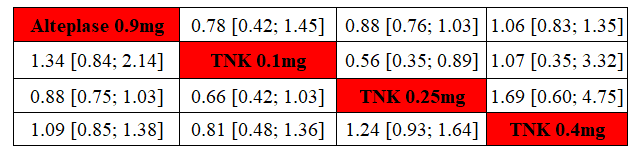
**

**Supplementary Figure S14: league table for Major neurological improvement within 72 hr**

**
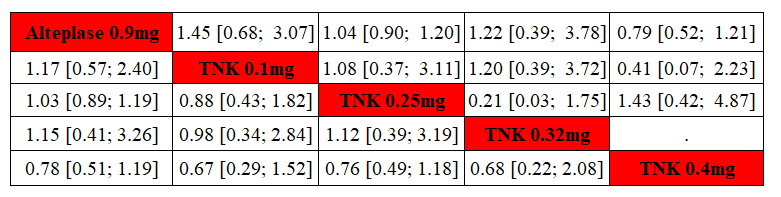
**

**Supplementary Figure S15: Mortality**

**
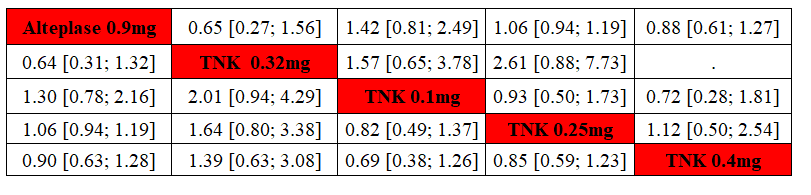
**

**Supplementary Figure S16: Poor functional outcome league table**

**
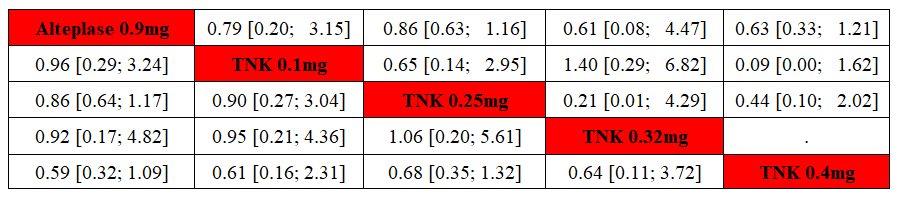
**

**Supplementary Figure S17: League table for Symptomatic Intracranial Hemorrhage**
